# Objectivity in science and law: A shared rescue strategy

**DOI:** 10.1016/j.ijlp.2019.02.004

**Published:** 2019

**Authors:** Matthew Burch, Katherine Furman

**Affiliations:** a University of Essex, School of Philosophy and Art History, Wivenhoe Park, Colchester CO4 3SQ, United Kingdom; b University College Cork, Department of Philosophy, College Road, Cork, Ireland

**Keywords:** Objectivity, Science, Law, Psychiatry, Epistemic risk, Phronetic risk

## Abstract

The ideal of objectivity is in crisis in science and the law, and yet it continues to do important work in both practices. This article describes that crisis and develops a shared rescue strategy for objectivity in both domains. In a recent article, Inkeri Koskinen (2018) attempts to bring unity to the fragmented discourse on objectivity in the philosophy of science with a risk account of objectivity. To put it simply, she argues that we call practitioners, processes, and products of science objective when they identify and manage certain important epistemic risks. We endorse this view and attempt to tailor Koskinen's strategy to the problem of objectivity in the legal context. To do so, we develop a novel notion of phronetic risk, and argue that we call practitioners, processes, and products of law objective when they identify and manage certain important epistemic and/or phronetic risks. Our attempt to rescue objectivity is especially important for work at the intersection of law and psychiatry. For that intersection represents a place where skeptical worries about objectivity in science and law work in tandem to pose serious critical challenges to contemporary practice; and our rescue strategy represents a promising way to negotiate those challenges.

## 1 Introduction

Objectivity is a central concern for many of the issues at stake in this journal; the intersection of law and psychiatry is a place where skeptical worries about objectivity in science and law work in tandem to pose serious critical challenges to contemporary practice. For instance, concerns about the scientific objectivity of psychiatry abound in the literature on mental disorders. Skeptics treat the term “mental disorder” as a purely evaluative term designed to justify medical intervention (Szasz, 1974); skeptics and non-skeptics alike point out that the diagnosis of mental disorders sometimes serves as a social tool of oppression that stigmatizes perfectly healthy but aneurotypical people (Eysenck, Wakefield, and Friedman, 1983); and non-skeptics who hold that mental disorders have an underlying physiological basis still insist that the concept has an ineliminable value component (Wakefield, 1992). Moreover, this evaluative dimension of the concept of mental disorder drives worries that the field of psychiatry is just not as objective as other areas of medicine. On the other side of this debate, however, defenders of psychiatry's objectivity point out that it operates within the scientific medical model (Shah & Mountain, 2007), and they argue that if we take interrater reliability as a measure of objectivity, “psychiatric diagnosis is often as objective as that in most other medical specialties” (Pies, 2007, p. 20). Concerns over objectivity in psychiatry thus pose two main questions: 1) is there such a thing as objective psychiatric disorder out there in the world; 2) if objective psychiatric disorder exists, can we objectively identify and measure it?

A similar back and forth plays out in the relevant legal discourse. The Committee on the Rights of Persons with Disabilities echoes the critics of psychiatry in its *General Comment No 1* (UN Committee on the Rights of Persons with Disabilities, 2014), when they claim, “Mental capacity is not, as is commonly presented, an objective, scientific and naturally occurring phenomenon (par. 14).” Moreover, many disability rights advocates argue that mental health law fails to be objective in that it reflects blatant bias against persons with intellectual and psychosocial disabilities (Harpur, 2009), arguing that the law takes cover behind the rhetoric of objectivity, while it stigmatizes and imposes “ableist” norms on persons with intellectual and psychosocial disabilities. But this skeptical viewpoint does not predominate. The European Court of Human Rights, for instance, specifies that the lawful deprivation of a person's liberty on the grounds that she is of “unsound mind” must be based on “*objective* medical expertise” and “*objective* medical evidence” (European Court of Human Rights, 2012 par. 103, emphasis added). Far from an idle theoretical question, then, objectivity is a central concern for contemporary public policy challenges at the intersection of psychiatry and the law.

Unfortunately, the concept of objectivity is in a crisis in science and the law. In both domains, the term is multiply ambiguous, referring to practitioners, processes, and products. In the scientific context, Heather Douglas (2004) has identified eight distinct meanings of process ‘objectivity’ alone, none of which is reducible to any other. And researchers at the Essex Autonomy Project have identified at least six distinct and mutually irreducible notions of objectivity in the law.^1^

This ambiguity has provoked searing deflationary critiques in both domains. To take a prominent example from the philosophy of science, Ian Hacking (2015) argues that objectivity is a useless “elevator concept” – by which he means a high-level philosophical concept, rather than a grounded one used by practitioners – and that we should stop talking about it. To do worthwhile work on issues traditionally discussed under the heading of objectivity, he contends, philosophers should do ground-level analysis that targets the specific epistemic ‘vices’ that afflict researchers in their everyday practice, such as bias or the corrupting influence of funding from interested third parties.

In the law, deflationary critiques have been even harsher. Catharine MacKinnon (1991), for instance, argues that “objectivity” has long served as a legal tool of oppression, because its lack of specific content allowed it to be co-opted as a stand-in for white male values. On her view, objectivity not only lacks cognitive content, but it functions as an instrument of injustice.

Despite these forceful deflationary critiques, the concept of objectivity continues to do important work in scientific and legal practice. In science, we rely on a division of epistemic labour, and objectivity allows that practice to persist. No one person can know everything, so we divide the epistemic tasks between us (Kitcher, 2011, pp. 21–22; Goldberg, 2011). To maintain this division of epistemic work, we need to trust that individual knowers and knowledge-production groups have done their tasks well, and that we can trust their results. Objectivity is the endorsement that we can trust the knowledge produced by others (Grasswick, 2010; Schemann, 2011) and thus allows us to continue this vital social practice. Scheman (2011) and Grasswick (2010) speak of larger audiences in the same terms: *laypeople* should be able to trust the outcomes of science. Objectivity assures them that such trust is warranted. The epistemic authority of science thus rests on its claims to objectivity (Koskinen, 2018; Reiss & Sprenger, 2017).

Objectivity serves similar functions in the law. The legal process also depends on a division of labour wherein different legal actors perform distinct tasks. For that process to work, judges, juries, expert witnesses and others must rely on each other to perform their tasks free from judgment-distorting factors like biases and conflicts of interests. Objectivity is the endorsement that we can trust them to have done so. Moreover, objectivity also enables the wider public to place its trust in the legal system. Hobbes (1651/1994) argued that the State exists so we can hand over our arduous individual claims to law enforcement to a third party. We need not endorse the Hobbesian account to accept that we participate in formal legal processes, rather than taking them on ourselves (like vigilante groups might), because we trust the legal system. In this vein, Postema (2001) argues that the law rests on legitimacy – on “the allegiance of all citizens” – and that this requires objectivity (p. 101). The normative practical authority of the law thus rests on its claims to objectivity.

In science and law, then, the concept of objectivity is ambiguous, subject to deflationary critiques, and yet continues to do important work. Given this shared predicament, we propose a shared rescue strategy. Specifically, we extend Inkeri Koskinen's (2018) risk account of objectivity in science and apply it to the law. Koskinen argues that we call practitioners, processes, and products of science objective – and so we trust them and believe that they warrant our trust – when they identify and manage certain important epistemic risks. This unifies the fragmented discourse on objectivity in the philosophy of science under the heading of epistemic risk management, and thus resolves the ambiguity that provides some of the fuel for deflationary responses. We endorse Koskinen's view and attempt to tailor it to the issues at stake in the legal context. To do so, we develop a novel notion of phronetic risk, and argue that we call practitioners, processes, and products of law objective when they identify and manage certain important epistemic and/or phronetic risks. Our hope is that this will likewise bring unity to an otherwise fragmented discourse, and, ultimately, help resolve the kinds of disputes we mentioned at the outset.

## 2 Koskinen's epistemic risk account

The ideal of objectivity has an ontological and epistemological aspect. The ontological aspect is concerned with the objective world, the world as it is unmarred by subjective distortions—an “absolute conception” of reality (Williams, 1985) grasped, so to speak, from a God's eye view, or “a view from nowhere” (Nagel, 1986). The epistemological aspect, on the other hand, deals with the normative standards that govern our efforts to know the world.

In keeping with a trend in recent philosophy of science to view science as a practice carried out by imperfect human agents for the sake of human ends and interests, much recent work on the ideal of objectivity sets the ontological aspect of objectivity aside. When you see science as a practice organized around the interests of imperfect human agents, the notion of “carving the mind-independent world at its mind-independent joints” starts to look overly ambitious (Elgin, 2017, p. 151). Setting the ontological question aside, this recent work focuses instead on constructing an epistemically normative ideal of objectivity via an analysis of the various ways actual scientists use the term “objectivity” in practice. We draw inspiration from Koskinen's (2018) important work in this vein.

Here are some of the ways Koskinen finds contemporary philosophers of science using the term objectivity: they treat objective claims as fallible; they think of objectivity not as an on/off concept but rather as something that comes in degrees; they allow for great variety in what can *be* objective, e.g., processes, people, claims, and communities; they deploy the term differently across disciplines, projects, and contexts; and their efforts to ensure objectivity likewise vary according to their current aims and context. To give a taste of this latter variety, Koskinen mentions three identified by Douglas (2004): 1) convergent objectivity—when researchers reach the same results via different means, 2) procedural objectivity—when a process allows for one researcher to be replaced by another without altering the result, and 3) interactive objectivity—when a research community fosters lively and diverse critical exchanges.

Next, Koskinen argues that we can unify this diversity with a “risk account of objectivity.” Her argument begins with a basic fact of human finitude: our imperfections as epistemic agents make our efforts to acquire knowledge subject to risks of error, i.e., epistemic risks. In science there are many important epistemic risks, but the ones we have in mind when we talk about objectivity are risks of error that we are profoundly and consistently prone to due to our frailties as epistemic agents, e.g., idiosyncrasies, illusions, cognitive biases, collective biases, and the like. Objectivity, according to Koskinen, assures listeners that they can rely on the products of science because “important epistemic risks arising from our imperfections as epistemic agents have been effectively averted” (p.1). Thus, on Koskinen's account, when a speaker calls a scientific practitioner or a piece of science objective, she indicates that at least one of these important epistemic risks arising from our imperfections as epistemic agents has been recognised and measures have been taken to mitigate it. This doesn't mean that objective science is infallible, just that best efforts have been made to avert certain factors that are likely to take us further away from the truth. Sometimes, in actual fact, scientists will still have gone wrong. However, it will still be your best bet to accept objective science, because it is more likely to be right than the alternatives, where these kinds of epistemic risks have not been managed. You should still rely on the science, even though it is fallible.

The risk account of objectivity captures many of the ways that historians (Daston & Galison, 2007) and philosophers of science (Douglas, 2004) have identified scientists using the term. Objective claims are fallible, because our attempts to manage epistemic risks can fail, e.g., we can overlook some epistemic risk, we can make an error in calculation, and so on. Objectivity comes in degrees because some practice P_1_ can mitigate epistemic risk R better than practice P_2_, while both practices do a pretty good job. There's great variety in what can be objective, because processes, people, claims, communities, and many other things can avert important epistemic risks arising from our imperfect epistemic agency. The way we describe and achieve objectivity varies significantly across disciplines, projects, and contexts, because different situations pose different epistemic risks. Finally, our efforts to ensure objectivity vary because different strategies avert different epistemic risks. For example, interactive objectivity – those communal aspects of scientific processes, such as peer-review and scientific debate – mitigate individual and collective sources of bias. Say an individual scientist has biased views on lung cancer that are fuelled by Big Tobacco funding. The practices that constitute interactive objectivity will subject his claims to multiple sources of critical scrutiny and so make it difficult for him get his biased views past the checks of the scientific community. According to Koskinen, what we see in the case of interactive objectivity applies to every type of objectivity identified by authors like Douglas: each one manages particular epistemic risks.

In this way, Koskinen unifies the fragmented discourse on objectivity in science under the heading of epistemic risk management, and she thereby rescues the scientific conception of objectivity from protracted disputes about its nature and merits. A similar rescue strategy is appropriate for objectivity in the law, but it requires that we think more expansively about the risks at play.

## 3 A risk account of objectivity fit for law

Much of the philosophical discourse on legal objectivity treats the law's objectivity as a question of its determinacy, maintaining that the law is objective insofar as it determines “uniquely correct outcomes” in actual or hypothetical cases (Brink, 2001, p. 65). Put otherwise, legal decisions are objective if and only if they achieve the result that the law *really* requires. This notion of ‘the one determinate correct answer’ serves as legal philosophy's analogue to the view from nowhere in philosophy of science. The best-known version of this approach is Ronald Dworkin's “right answer thesis” which maintains that most legal cases – even hard cases – have objectively correct determinate answers. And, as Dworkin (1986) argues in *Law's Empire* and elsewhere, the correct answer to a legal dispute offers the best fit with the law's institutional history and the best moral justification of that history. Thus, for Dworkin, objectivity in law depends not just on the objectivity of legal interpretation but also on the objectivity of morality, i.e., “an independent, subsisting realm of moral facts” (Dworkin, 1996, p. 97).

Other legal philosophers defend the view of legal objectivity as determinacy, while rejecting the link Dworkin forges between law and morality. Brian Leiter, for example, argues that if we tie objectivity to moral realism, few will buy it, because an absolute conception of morality is a hard sell. Leiter still insists that an objective conception of law requires determinate answers, but he proposes a naturalistic alternative to Dworkin's approach. According to Leiter, we should base our determinate correct answers to legal questions not on non-natural moral facts but rather on a “metaphysically objective” realm of “legal facts” (Leiter, 2002, p. 969) that, like the objects of nature, are “objective in the sense of being ‘mind-independent’ and causally efficacious” (Leiter, 2001, p. 67).

Whether it's grounded in Dworkin's moral realism or Leiter's naturalism, this approach to legal objectivity strikes us as overly ambitious. If legal objectivity demands that we attend faithfully to the mind-independent moral and/or legal facts to determine uniquely correct answers to legal disputes, then it demands too much. Why think that there's a uniquely correct determinate answer to nearly every legal question? The law, after all, is a contingent and frequently messy historical practice organized around the evolving interests of imperfect human agents; and the situations to which we apply the law are often just as complicated and messy. Moreover, to understand objectivity as determinacy is to treat it as an on/off concept—you either determine the uniquely correct answer to the legal dispute, or you don't. And this leaves no room for the sort of comparisons, familiar to legal practice, between more and less objective decisions, judges, and procedures. Moreover, it rules out the possibility that legal actors can make judgments that are both objective *and* fallible. Like the view from nowhere in the philosophy of science, then, this looks like objectivity fit for gods, not limited imperfect agents engaged in a practice organized around specific cultural and historical human interests.

Indeed, critics have reacted to the determinacy view of objectivity precisely along these lines. For instance, authors from Critical Legal Studies like Roberto Unger (1986) have argued that if objectivity requires determinacy, then objectivity is beyond our reach, because there's always room for rational indeterminacy when we seek to apply an abstract body of law – in light of its institutional history – to the facts of a particular case. Many scholars and legal practitioners agree: if objectivity in law depends on a) the existence of mind-independent moral and/or legal facts and b) our ability to know those facts, then it's an unattainable ideal and we would be better off if we just stopped talking about it.

We propose another way forward. We agree with Leiter that we should take our cue from the natural sciences when developing a concept of legal objectivity (2001, p. 67); but our agreement stops there, because we think Leiter latches on to the wrong model of scientific objectivity. Instead of pursuing an analogue to the view from nowhere, we should follow the lead of more recent work in philosophy of science. We should set aside ontological questions about some objective realm of moral and/or legal facts and concentrate instead on developing an epistemically and practically normative account of objectivity. And we should develop that account not in terms of some abstract conception of the law but rather in relation to the ways actual legal practitioners use the term objectivity. To develop such an account, we adapt Koskinen's risk account of objectivity to the legal context.

Given her interest in scientific objectivity, Koskinen naturally focuses strictly on the epistemic risks that objective science averts. But objectivity isn't a purely epistemic ideal. It also plays a role in a range of other contexts – e.g., morality, politics, and law (Nussbaum, 2001; Shafer, 2003; Sibley, 2001) – where the stakes are decidedly non-epistemic. So when we talk about legal objectivity, we mean something broader than epistemic risk management.

How should we think of the non-epistemic risks posed by the legal context? Biddle and Kukla (2017) provide a clue. Although they also focus exclusively on knowledge production, Biddle and Kukla identify a broad range of risks that are not strictly epistemic but nevertheless bear on our epistemic practices. Philosophers of science have long-accepted that moving from a body of evidence to a hypothesis involves risk – the so-called problem of ‘inductive risk’ (Rudner, 1953). Biddle and Kukla argue further that risk in knowledge production is more ubiquitous, involving everything from model selection through to how particular phenomena are classified. They describe this more expansive notion of risk as ‘phronetic risk’, which they define as “epistemic risks that arise during the course of activities that are preconditions for or parts of empirical (inductive or abductive) reasoning” (p. 220). Such risks arise, then, when researchers must deliberate in light of their values and interests in order to make calls that are fateful for the balance of epistemic risks.

We want to expand this category of phronetic risk to tailor Koskinen's risk account of objectivity to the legal context. When we speak of objectivity in the law, we are most often concerned not just with accurate knowledge acquisition strategies but rather with the normative requirements of practical reason. Consider a few examples. We claim that divorce proceedings are objective when the legal decisions that determine the outcome treat all relevant parties fairly; objectivity here thus averts the risk of our practical reasoning failing to track the normative requirement of fairness that we associate with justice. To take another example, we say that a judge's decision fails to be objective when it's shaped by racial bias; objectivity in his case would have averted the risks of personal bias and racial animus affecting his reasoning and decision-making. Finally, when a witness on Big Pharma's payroll testifies to the moral integrity of the CEO who runs her company, we might doubt her objectivity, as objectivity on her part would require her to prevent a considerable conflict of interests from distorting her appraisal of her boss's character. In these examples, we see legal actors running the risk of getting things wrong, but the risks at play, at least principally, are not epistemic. For the most part, we're not worried that an attempt to produce knowledge will go wrong in these cases; rather, the relevant risks represent threats to practical reasoning. These examples are undoubtedly concerned with *phronetic risk,* then, but not of the sort of *epistemic*-phronetic risk that Biddle, Kukla and Koskinen have in mind. These examples from the law are not targeting a sub-category of epistemic risks, but rather a class of risks that are strictly phronetic, i.e., risks of getting things wrong in our practical reasoning.

We thus need to extend the notion of phronetic risk beyond epistemic concerns to cover the wider range of risks encountered in the practical reasoning of the legal system. To articulate this broader notion of phronetic risk, we turn briefly to Aristotle's *Nichomachean Ethics*. In that work, Aristotle identifies five intellectual virtues. One of them is epistēme, which, as most readers will know, is typically translated as knowledge (or scientific knowledge), and from which the term epistemic derives. Hence, epistemic risks threaten our efforts to acquire knowledge. Another intellectual virtue Aristotle names there is phronēsis, which is typically translated as practical wisdom or practical intelligence, and from which we derive the term phronetic. Phronēsis does a lot of important work in Aristotle's virtue ethical theory, but we will narrowly tailor our discussion of the concept to our current concerns.

Phronēsis denotes the human capacity for “concretely situation-specific discernment” (McDowell, 2007, p. 340). According to Aristotle, without this capacity one cannot live a virtuous life. For, he argues, even if we get lucky, and we're naturally endowed with virtuous impulses, and our upbringing habituates us to seek the good and teaches us our culture's code of conduct, without phronēsis, we will still be apt to go wrong with respect to the demands of virtue. Why? Because doing the right thing isn't simply a matter of rule-following; as Aristotle (2000) famously argues, right action requires that we act “at the right times, with reference to the right objects, and to the right people” (110b21–22). Think of it this way. You can teach a child with a good disposition and good habits that justice is “the constant and perpetual will to render each his due” (*Institutes of Justinian*)^2^; but you cannot thereby expect the child to know how to render each person her due across the vast range of diverse and evolving concrete circumstances that life presents us with. Such situation-specific discernment requires the practical intelligence to a) move from general rules to specific circumstances; b) descry the morally salient features of novel and ever-shifting situations; c) discern which course of action (or set of actions) the available reasons best support; and d) discover and know how to secure efficient means to achieve that course of action (or set of actions). And she must do all this in a way that is sensitive to and constrained by the relevant moral considerations. This is the work of phronēsis. It is the capacity to make context-sensitive calls about the best course of action across a range of variable, dynamic concrete circumstances.^3^ Such calls are risky, because even the most skilful practical reasoners can get them wrong. Moreover, going wrong in such cases is not a matter of failing to formulate accurate knowledge claims; rather, it's a matter of failing to discern what the situation calls for morally and practically speaking. Thus, the risks that threaten our ability to get this sort of call right are not epistemic but rather phronetic.

If epistemic risk is “any risk of epistemic error that arises anywhere during knowledge practices” (Biddle & Kukla, 2017, p. 218); phronetic risk, as we conceive it, is any risk of error that arises during practical reasoning about the correct judgment to make and the best course of action to take. Just as epistemic risks represent a hurdle to science achieving its theoretical aims, especially its paramount aim of truth, phronetic risks pose a hindrance to the law achieving its guiding practical aims, especially its highest aim of justice. Moreover, just as objective science continually faces and mitigates diverse epistemic risks, objective legal practice continually faces and mitigates diverse epistemic and phronetic risks.^4^ Finally, in the scientific context, we saw that objectivity is concerned with managing important epistemic risks that we're subject to profoundly and consistently because they arise from our imperfections as knowers. The same is true in the case of phronetic risks: the ones objective legal practice strives to avert arise from our imperfections as practical reasoners.

As knowers and deliberators, we aim to get things right in theory and practice. To do so, we must avert the important epistemic and phronetic risks that stand in our way. Some of these important epistemic and phronetic risks arise from our imperfections as epistemic and practical agents. Objectivity is the assurance that at least some of these latter risks have been managed.^5^

With this expanded account of phronetic risk in view, we propose that objectivity in the law is the assurance that measures have been taken to manage the epistemic and phronetic risks of error inherent in legal practice. Objectivity is a self-responsible stance that strives to identify and avert epistemic and phronetic risks that arise from our imperfections as agents. Such a risk account of legal objectivity will have all the virtues of its scientific corollary. First, it allows for objective legal claims to be fallible, which is attractive given the inherent fallibility of human judges and other legal actors. Second, it entails that objectivity is not an on/off concept but rather comes in degrees, which allows us to compare judges, procedures, and outcomes along a continuum of objectivity. Third, it allows us to account for the great variety of things in the law that are said to be objective: evidence; expert witnesses; judges and their decisions; the jury and its verdicts; procedures; standards; legislative bodies; and the legal system itself. These things can all be objective, but they are so in their own way, because they avert different epistemic and/or phronetic risks. Finally, this approach allows us to tailor our understanding of what objectivity demands to our current aims and context, because the epistemic and/or phronetic risks we face will vary with our aims and context.^6^ Importantly, the language of ‘phronetic risk management’ is not intended to imply anything approximating a ‘check boxing’ approach to risk, or any managerial bureaucratic oversight. Rather, drawing on our earlier discussion of Aristotle, we conceive of this as a type of practical wisdom.

In the second half of this paper, we change pace somewhat. We follow Koskinen's procedure by identifying some of the products, persons, and processes that actual legal practitioners deem objective and indicating which epistemic and/or phronetic risks they avert. There are undoubtedly more types of objectivity and associated risks in the law than we will identify here, and still more will likely emerge in the future. After all, as Koskinen notes, the risks a community deems salient change over time. Our aim here is not to offer an exhaustive list, but rather an indicative one that illustrates how a risk account of objectivity would function in the legal context. We did not, however, construct our list at random; rather, we selected certain types of objectivity and epistemic/phronetic risks due to their prevalence in current legal practice. Indeed, we think it's hard to imagine how a legal system could make claims to objectivity without managing the epistemic and phronetic risks we discuss below. To continue our comparison between scientific and legal objectivity, we will introduce each type of legal objectivity with a quick look at its scientific analogue.

## 4 Types of objectivity and the risks they manage

### 4.1 Hermeneutic objectivity

The contemporary philosophical concern with epistemic risk management began with the problem of inductive risk, i.e., the problem of moving from a body of data to a unique interpretation (Rudner, 1953). That move involves potential ethical, phronetic, and epistemic risks. For example, imagine a public health context where accepting hypothesis H_1_ might lead to 1000 deaths, while accepting H_2_ might lead to 1000 people experiencing slight discomfort. Accepting either hypothesis involves ethical risks, i.e., risks of harm, but we would demand a higher degree of certainty to accept H_1_ because the ethical stakes are higher. Moreover, the move from data to interpretation in this case also involves managing phronetic risks, because we have to make a practical moral judgment about the balance of those ethical risks, and we could get that call wrong. Finally, there are also epistemic risks to consider. The very fact that we are not dealing with deduction but rather making an inductive move that involves degrees of probability makes us vulnerable to the risk of epistemic error. One of the ways objective science manages such epistemic risks is via interpretative norms. For instance, consider the standardisation of p-values, i.e., the standard that 5% significance is required to reject the null hypothesis and accept your own hypothesis. As Parascandola (2010) argues, such p-value standardisation is an epistemic risk management strategy.^7^ The scientific community agrees on a standard p-value to offset worries about uncertainty in each new case.

An analogue to inductive risk runs through the practice of legal interpretation. When judges decide a case, for instance, they must bring legal norms, moral principles, and the law's institutional history to bear on the concrete particulars of a case. Like the move from data to interpretation in science, this hermeneutic move from the law to the facts of the case is shot through with uncertainty. Not only is there often a low probability of a unique, determinate, and correct interpretation of a case in light of our legal norms, moral principles, and the actual institutional history of the law. But it is even less likely that an imperfect limited agent will actually know what that uniquely correct interpretation is. In the words of former Chief Justice of England, Lord Bingham, “when you are deciding a case you usually feel that there is a choice of answers… …To say that there is one right answer and one wrong answer is just not at all how it feels.”^8^ This uncertainty makes the move from the law to the particulars of a case risky. The move almost always involves ethical risks – i.e., risks of harm to others – because legal decisions tend to have real-world consequences. Moreover, moving from the body of law to a particular case also always involves phronetic risks: we can go wrong with respect to the normative practical requirements of the law. For example, there's the phronetic risk of interpreting the law in a way that leads to an unjust decision, or a decision that is less just than a viable alternative would be.

Objective judges acknowledge these phronetic risks inherent in the hermeneutic process and take steps to avert them. This includes relying on techniques that are analogous to the interpretative norms of science just discussed (Eskridge & Ferejohn, 1994; Knight & Johnson, 1994). One example of an interpretative strategy designed to mitigate phronetic risk is the principle of *stare decisis*, or the requirement that judicial precedent be given interpretative priority. When judges prioritise precedence in this way, we have more reason to trust them to interpret the law in similar ways in similar cases (Foster, 2008). This averts an obvious ethical risk—for it would be unjust to hold people accountable to unpredictable laws. But the practice also manages phronetic risks, e.g., when judges are tempted to make idiosyncratic calls or to “legislate from the bench” on the basis of pet ideologies, the practice of prioritising precedence anchors their reasoning in the common law and a shared history of interpretation that tempers their personal take on things. Again, this is an instance of phronetic, rather than epistemic, risk management—we don't expect that adhering to precedent will get us closer to the ‘true’ interpretation of the law, only that it guards against risks like idiosyncrasy and individual bias.

### 4.2 Procedural objectivity

Earlier we touched on procedural objectivity in science, i.e., when a process allows for one researcher to be replaced by another without altering the result. With procedural objectivity, scientists rely on methodological procedures to avert the epistemic risks of idiosyncrasy and individual bias. Daston and Galison (2007) famously describe ‘mechanical objectivity’ along these lines:

By *mechanical objectivity* we mean the insistent drive to repress the willful intervention of the artist-author, and to put in its stead a set of procedures that would, as it were, move nature to the page through a strict protocol, if not automatically

(Daston & Galison, 2007, p. 121).

The idea, then, is that such procedures eliminate distortions that stem from the scientist's own subjective contribution to her observations and thereby enhances her focus on the objects she observes. Moreover, the fact that any other scientist could follow the procedure and attain the same results suggests that it successfully manages the epistemic risks associated with subjective distortions like idiosyncrasy and individual bias. If multiple researchers reach the same result, then it's less likely that any idiosyncrasies or biases specific to them as individuals have distorted that result. Finally, strict protocols also prevent the scientist from inadvertently introducing a change to the process that affects the outcome.

In the law, procedural objectivity plays an analogous role. Some legal procedures principally aim to manage epistemic risks. The Law of Evidence, for instance, focuses on averting epistemic risks by ensuring that legal actors follow appropriate procedures in acquiring the evidence that appears before the Court. As Alvin Goldman (2005) notes, it's generally held that one of the principal aims – if not *the* principal aim – of evidence-handling procedures is to promote “the accurate or truthful determination of facts relevant to the case at hand” (p.164). For example, proper procedures of evidence management require that relevant parties handle evidence in a way that leaves no significant room for doubt that someone could have accidentally altered or deliberately tampered with the evidence. The analogy to procedural objectivity in science is clear. Just as a strict experimental protocol is designed to prevent an individual scientist from introducing changes to the process that affect the results, following a strict evidence-handling procedure in criminal cases assures us, to a reasonable degree, that no one has corrupted or substituted the evidence in a manner that leads us away from a truthful determination of the facts. In our terms, then, these procedures contribute to the law's objectivity by helping us avert important epistemic risks.^9^

Other parts of the law use procedures predominantly to manage phronetic risks. The proceduralisation of the judicial process aims to allow for one legal actor to be replaced by another without significantly altering the result. This provides a useful lens to understand procedural rights and our highly proceduralised court hearings. For example, consider some of the procedural characteristics that Jeremy Waldron (2011) deems indispensable to the rule of law. According to Waldron, the government should not impose any “penalty, stigma or serious loss” on someone without procedures that involve:

A. A hearing by an impartial tribunal that is required to act on the basis of evidence and argument presented formally before it in relation to legal norms that govern the imposition of penalty, stigma, loss etc.;
B. A legally-trained judicial officer, whose independence of other agencies of government is assured;
C. A right to representation by counsel and to the time and opportunity required to prepare a case;
D. A right to be present at all critical stages of the proceeding (2011, p. 6).^10^

These procedural characteristics clearly aim to manage the phronetic risks of making biased, discriminatory, and/or arbitrary decisions. The basic idea is that no matter who you are, and whatever your personal characteristics, the law's procedures exist to ensure that you receive the same treatment as anyone else not relevantly different from you. In other words, such procedures are designed to prevent legal actors from imposing their idiosyncrasies and biases on the legal proceedings. In both the scientific and the legal context, then, the emphasis on procedures is a way to manage the epistemic and/or phronetic risks that individual practitioners might intentionally or inadvertently contaminate evidence or impose their personal quirks and biases on the process.

### 4.3 Objectivity as independence of judgment

Another strategy for managing epistemic risk in science is the attempt to maintain independence of judgment. It's well known that funding from interested third parties tends to skew research results towards those parties' interests; and, correlatively, we also know that the best evidence tends to come from studies conducted by scientists who operate independently of such interested parties. For example, as Stegenga (2018) notes, the most accurate and reliable randomized controlled trials and systematic reviews to test medical interventions are typically carried out by “academics who are independent of the manufactures of the medical interventions in question” (p. 2). When researchers have an interest in the outcome of scientific research, they tend to err on the side of that interest, which quite often takes them away from the truth. The scientific community thus promotes strategies designed to avert this epistemic risk, such as requiring researchers to declare their funding sources and other conflicts of interests. These strategies aim to mitigate idiosyncrasy, bias, and motivated reasoning, and to encourage other members of the scientific community to closely scrutinise the research results.

The law also treats independence of judgment as an essential strategy to manage phronetic risk. Notice what Waldron puts second on his list of indispensable procedural characteristics: a “legally-trained judicial officer, whose independence of other agencies of government is assured”. This aims to assure the person before the law that the outcome will not be predetermined by third party interests that are normatively irrelevant to the adjudication of her specific case. But independence of judgment does not just require distance from the interests of third parties; it also requires that persons involved take measures to prevent their own personal interests from affecting their judgment. We can understand anti-sympathy instruction for jurors in this light. For example, consider California v. Brown (1987) wherein the trial court instructs jurors not make their decision based on “mere sentiment, conjecture, sympathy, passion, prejudice, public opinion or public feeling.” We see a similar concern addressed by the Court of Protection of England and Wales in *CC v KK and STCC* (2012), as Justice Baker writes,

[T]here is a *risk* that all professionals involved with treating and helping that person – including, of course, a judge in the Court of Protection – may feel drawn towards an outcome that is more protective of the adult and thus, in certain circumstances, fail to carry out an assessment of capacity that is *detached and objective*. On the other hand, the court must be equally careful not to be influenced by sympathy for a person's wholly understandable wish to return home (par. 25, emphasis added).

This passage identifies two potential personal interests – the urge to protect a person and sympathy for her wishes – that, in our terms, pose phronetic risks that judges and others involved in such cases must manage with what Justice Baker characterizes as a detached and objective stance. Again, independent judgment requires independence not just from the interests of third parties but also from one's own interests, however noble or sensitive their underlying motives may be.

Whether motivated by group-interest or self-interest, conflicts of interest are inimical to independence of judgment, as they motivate the agent to decide on behalf of her group or herself, rather than attending to normatively relevant facts, reasons, and arguments. If a judge has a personal or social stake in the outcome of a case, those involved might naturally worry about her potential lack of impartiality.^11^ If a juror can't regard the defendant without prejudice, then the latter will not be able to trust the former's reasoning and ability to follow the evidence where it leads. Procedures, protocols, and instructions designed to promote independence of judgment thus contribute to the law's objectivity by managing the phronetic risks of partiality and conflicts of interest that could lead the process of legal reasoning away from a normatively correct judgment.

### 4.4 Objectivity as publicity, or deliberative objectivity

Earlier we mentioned interactive objectivity in science, which obtains when a research community fosters lively and diverse critical exchanges. This type of critical, argumentative activity aims to mitigate risks of idiosyncrasy as well as individual and collective biases (Longino, 1990). It's clear enough how critical dialogue manages idiosyncrasy and individual bias: it allows the community to identify and eliminate merely personal takes on the available evidence. How a critical exchange uncovers collective bias, however, is less obvious. Collective biases, after all, are shared, and so they tend to operate as background assumptions that shape the group's conversations; however critical their exchanges may be, then, it's unclear what mechanism could reliably bring such shared background assumptions into view.

This highlights the importance of outsiders for interactive objectivity, a point that has gained theoretical prominence with the development of Standpoint Theory in Feminist Philosophy of Science. Standpoint theories, such as Harding (1991) and Wylie (2003), hold that the “view from nowhere” or the so-called “value-free ideal” is impossible. Instead, they argue that all knowledge is from a particular perspective and advocate for more diverse scientific communities, because looking at the evidence from different social and political perspectives may allow for different factors to become more or less salient (Wylie, 2003). As an example of this, consider the case of early AIDS science as described by Steven Epstein in *Impure Science* (1996). AIDS was initially aetiologically mysterious, but the initial framing of AIDS was that it must be caused by some aspect of the ‘gay-lifestyle’ (rather than being an infectious disease with a microbial cause), focusing in on the most sensationalised aspects of the lifestyles of men in the ‘urban American gay-scene’ (e.g., promiscuity and drug use), and thus dismissing evidence that ran counter to the hypothesis that it must be life-style related; such as the monogamous gay men, heterosexual men and women, and children who presented with AIDS (Epstein, 1996, pp. 48–50). Ultimately it was microbiology and the discovery of the HIV virus, not diversifying the scientific community, that put us on the right causal track, but this provides a cautionary tale of the dangers of looking at the evidence from one social/political perspective. Thus, the example constitutes a negative illustration of the importance of interactive objectivity. Consider a related positive illustration: later in the 1980s, when treatment first became available, mainstream science had to consult members of the AIDS activist community, because they had become the experts, as those living with the disease, and this perspectival knowledge was essential for successfully developing treatment (Epstein, 1996).

In the legal context, the analogue to interactive objectivity is what legal philosophers call ‘objectivity as publicity’ or ‘deliberative objectivity.’ On Postema's (2001) compelling account of this form of objectivity, a judgment is objectively correct if and only if it can be justified by public practical reason. The basic idea is that deliberative objectivity holds for practical reasoning procedures and their outcomes when they are acceptable to all (in practice or in principle). Thus, we achieve deliberative objectivity when we arrive at judgments through a public, deliberative process that fully considers and assesses the available and normatively relevant arguments and reasons. Under the best circumstances, we can see this kind of deliberative objectivity at work in the jury process, law consultations, and legal argumentation.

Like interactive objectivity, deliberative objectivity aims to mitigate risks of idiosyncrasy as well as individual and collective biases. To do so effectively, however, accounts of deliberative objectivity in the law need to take a cue from Feminist Philosophy of Science and its emphasis on perspectival diversity. To achieve a truly robust deliberative consensus, it's not enough that a claim be “maximally supported by the arguments and the balance of reasons available for articulation and assessment by reasonable and competent persons in a fully public deliberative process” (Postema, 2001, p. 117). Such maximal argumentative support is not enough because which reasons are “available for articulation and assessment” hinges crucially on which people we include in the deliberative process. Again, critical exchange might eliminate idiosyncrasy and individual bias, but it's unlikely to root out collective bias unless the group hears the voices of outsiders. Otherwise, the homogeneity of legal practitioners can obscure collective biases at work in the legal system.

Consider, for example, the case of *Buck v. Bell* (1927) in which the United States Supreme Court upheld a Virginia circuit court ruling that the state should sterilise Carrie Buck, a young, poor, single mother, falsely alleged to be “feebleminded.” At the time, the Supreme Court had several enthusiastic supporters of eugenics on the bench, including Oliver Wendell Holmes, who wrote the Court's opinion. Under such conditions, a critical evaluation of *available* reasons and arguments falls short. Had the conversation included the voices of women, people living in poverty, and/or people with disabilities, the Court's collective bias may have been exposed. Maximal argumentative support is not enough; diversity of perspectives is also essential to making objectivity as publicity properly public. Of course, such diversity will never guarantee the elimination of collective bias—such a conceit would only lull us back into the dreamy stupor of the illusory view from nowhere. But the voices of outsiders remain our best hope for catching sight of the blind spots that lie hidden in consensus views. This captures some of the impetus behind the well-known slogan and principle of disability rights activism, “Nothing About Us Without Us”; many activists believe, rightly we think, that conversations about disability rights will be inflected with collective biases that persons with disabilities are uniquely positioned to identify, challenge, and correct.^12^

Despite the analogy between interactive and deliberative objectivity, we should highlight the fact that the former principally manages epistemic risks, while the latter mitigates phronetic risks. That is, the former helps us avoid error in knowledge production, while the latter, as the Buck case illustrates, manages the risk of our practical judgments falling short of the requirements of justice.^13^

The intuition behind deliberative objectivity is clear. It rests partially on the notion that one's judgments improve when they must be defended against critical scrutiny, and partially on the intuition that one of the virtues of objectivity is transparency. Even Munro and Hardie (2018), in their criticism of objectivity, accept that the transparency associated with the term is worth striving for, because it allows others to check that no biases, idiosyncrasies or malevolent motives have warped the reasoning process.

### 4.5 Structural objectivity

Like interactive and deliberative objectivity, structural objectivity also manages a group-level phronetic risk, but not one that diverse critical exchanges can do much to manage, i.e., ‘structural biases’. Structural epistemic biases refer to the ways in which the very structure of the scientific research environment leads us to neglect the perspectives, stories, and interests of large swathes of society and thereby skews our knowledge base. To be clear, this is not a problem that is necessarily solved by just making the research community more diverse: diverse scientific research communities can (and many do) work in structurally biased research environments. Consider, for example, the case of neglected diseases, as discussed by Reiss and Kitcher (2009). Neglected diseases are those that receive a proportionally smaller share of the available research and biomedical resources relative to their contribution to the global disease burden. Note:

For instance, malaria, pneumonia, diarrhea and tuberculosis together account for 21% of the global disease burden, but receive only 0.31% of all public and private funds devoted to health research

(Reiss & Kitcher, 2009, p. 264).

The real-world consequence of this is that many thousands of people die from diseases that almost exclusively afflict the poorest parts of the world, many of which have been completely eradicated from more affluent regions. Tuberculosis, for instance, results in 1,566,000 deaths annually, despite being all-but-eliminated from richer portions of the world (although, it is still a problem amongst socially and economically marginalised segments of wealthy countries, such as the United Kingdom). One major reason that biomedical research neglects diseases that mostly affect the poor is that large pharmaceutical companies undertake the majority of that research. The cost of taking a new drug from initial research to shelf is astronomically large (around $800 million for a single new drug). Since pharmaceutical companies could never expect to make back this investment in the world's poorest regions, they do not undertake research that is relevant to these places (Reiss & Kitcher, 2009, pp. 265–267). This is a structural problem that is unlikely to be addressed by the influx of researchers from more diverse backgrounds.

In addition to being an obvious ethical concern, this poses an epistemic risk, in that we end up with medical knowledge skewed towards one very small segment of the world's population. Moreover, interactive objectivity can't manage this risk—introduce scientists from diverse backgrounds into this field of medical research and there still won't be money for research on neglected diseases. The main safeguard against this kind of epistemic risk is structural objectivity—the attempt to change the research environment so that it's not systematically skewed towards the perspectives, stories, and interests of privileged members of society. Reiss and Kitcher (2009) suggest that the way to do it in the case of neglected diseases is to implement Kitcher's suggestions for ‘well-ordered science’, as outlined in his book *Science, Truth and Democracy* (Kitcher, 2001). The details of this suggestion are too lengthy for us to spell out here, and not directly relevant to our ends. The point is that philosophers of science take seriously the risk of structural bias, like that seen in the case of neglected diseases, and they have suggestions for how we might manage that risk.

In the law, we see an analogous strategy at work, with feminist philosophers once again leading the way. Prominent feminist critiques argue that the law is not objective because it exhibits structural biases, systematically subordinating the interests of women to those of men (Mackinnon, 1983, 1987). Mackinnon, for instance, argues that the ideal of objectivity is in fact the male point of view masquerading as a “nonsituated, universal standpoint” (1983, p. 636). The basic claim behind her critique is that the very structure of the law privileges powerful white men and disadvantages women, ethnic minorities, persons living in poverty, and persons with disabilities; and the law deploys the rhetoric of objectivity to cover over this systemic oppression. If this legal order is simply what objective, value-neutral reason demands, then it cannot be unjust. In this way, Mackinnon argues that the language of objectivity “reinforce(s) existing distributions of power” (1983, p.645). Authors working in Critical Legal Studies (Unger, 1986), Critical Race Theory (Crenshaw, Gotanda, and Peller, 1995), and Disability Studies (Wolbring, 2008) have similarly argued that the law is not just structurally sexist but also racist and ableist. Some think this critique recommends the view that the very ideal of objectivity is corrupt; but others see it as a call to a deeper and more adequate notion of objectivity.

People who fight for structural objectivity in the law attempt to change how the law works, so that it doesn't systematically favour privileged groups of people. On this view, then, the law exhibits structural objectivity to the extent that it treats everyone the same unless they are relevantly different.^14^ Why is this a matter of objectivity at all and not strictly an ethical issue of non-discrimination? The basic idea is that a discriminatory legal system is shaped in light of – and so biased towards – a privileged form of subjectivity. Moreover, just as a structurally biased research environment will skew our body of knowledge towards the interests of privileged groups, so will a structurally biased legal system skew our body of legal judgments towards the advantage of some groups and the disadvantage of others. Whereas a legal system wherein “all persons are equal before and under the law and are entitled without any discrimination to the equal protection and equal benefit of the law” (UN General Assembly, 2006, Article 5.1.) is objective in the sense that it is not biased towards the interests of a particular form of subjectivity, and so it should not produce a body of judgments that systematically helps certain groups and hinders others. An objective legal system roots out structural biases and treats everyone the same regardless of their personal characteristics, and by doing so, it manages some of the deepest risks that legal reasoning will run afoul of the demands of justice.

### 4.6 Objective evidence

Finally, we turn to “objective evidence”, a common expression in scientific and legal discourse. Although the nature of evidence is a major philosophical issue in its own right, we only wish to sketch the link between objective evidence and epistemic/phronetic risk management.

First, note the basic ambiguity in the scientific use of the expression “objective evidence”. Sometimes we use the expression as if it refers to a particular kind of evidence; as though the evidence were objective by its very nature. What we typically mean by this is that the evidence is publicly available.^15^ Such evidence is objective, in our sense, because its public availability manages certain epistemic risks. Because the evidence is out there for all to see, we can come at it from different perspectives and appeal to it as a neutral arbiter when adjudicating disputes. And this intersubjective triangulation on a piece of public evidence mitigates the epistemic risks of idiosyncrasy and personal blind spots. To be clear, we are not just talking about procedural or interactive objectivity again. It's not the researchers' activity alone that makes the evidence objective. There's something about the evidence itself that makes it objective, namely, its publicity. Such evidence is objective in a way that, say, purely private mental experiences are not. Multiple people can see public evidence from different perspectives, and so the very nature of such evidence mitigates the risk of certain kinds of error.

But we also speak of objective evidence in at least one other way: we refer to the results of scientific inquiry – the products of science – as objective evidence. For example, when asked for objective evidence for the claim that X causes Y, we will likely appeal to empirical research results meant to demonstrate that causal link, e.g., “You want objective evidence? Here are 30 RCTs linking X to Y.” In this case, as Elgin (2017) puts it, “What justifies calling a particular result objective is that it is the product of an objective procedure” (p. 159). In our terms, the results of scientific inquiry can count as objective evidence in favour of some claim, because those results were reached by processes that avert important epistemic risks. What sort of processes? Exactly the sort we've discussed thus far: lively critical exchanges, peer review, triangulation, practices that root out conflicts of interest, critical scrutiny of collective and structural biases, strict experimental protocols, and so on. Again, we're not just repeating what we've said about other forms of objectivity here; rather, we're pointing out that results reached via these various strategies of epistemic risk management earn the label of objective. Objective evidence in this second sense denotes results we can rely on because they were reached via strategies that manage epistemic risk; and we should rely on those results to a degree that matches the rigor and known reliability of those risk management strategies. Thus, we have i) evidence that is objective in virtue of its public nature and ii) evidence that is objective in virtue of the epistemic risk management strategy by which it was reached.

Objective evidence in the law reflects a similar ambiguity. Much like the scientific context, the preferred and most powerful form of evidence in legal practice is publicly available evidence. For example, in criminal proceedings, prosecutors tend to prefer DNA evidence that places the defendant at the scene of the crime to the testimony of an eye-witness. Eye-witness testimony is replete with well-known epistemic risks, e.g., racial bias, conflicts of interest, the unreliable and creative nature of human memory, and outright lying; whereas hard physical evidence is publicly available, out there for everyone to see. The prosecutor can tell the jury, “The objective evidence is right before your nose. All you have to do is look.” Physical evidence is objective, then, in the sense that relying on it averts, at least to some degree, epistemic risks like personal bias, conflicts of interest, fallible human memory, and dishonest testimony. The very publicity of physical evidence makes it objective, although this by no means implies that such evidence can't lead us astray. It's simply to say that there are reasonable grounds to prefer it to less reliable alternatives.

Again, as we saw in the scientific context, evidence in the law is also called objective in virtue of the process by which it is acquired. For example, law consultations on matters of public policy invariably involve the review of relevant scientific evidence. Consider, for instance, the final report from the Wessely Review – an independent review of the Mental Health Act (1983) commissioned by the UK Department of Health – which states that, on top of a robust consultation process, “we spent time assessing existing evidence and data in relation to the Mental Health Act, and commissioned bespoke data analysis to inform our findings” (Department of Health and Social Care, 2018, p. 41). Moreover, the report assures us that not only was the existing evidence considered, but a premium was placed on evidence reached via the most rigorous available scientific methods. And this is all meant to assure the public that the evidence they relied on was objective—that it was reached via methods that manage important epistemic risks.

Finally, to drive home the difference between the two types of objective evidence we've discussed, consider an example that highlights the distinction. Say that the court has a choice between E_1_ and E_2_. E_1_ is physical evidence acquired in accordance with the proper evidence-handling procedures we discussed in §4.2; and E_2_ is physical evidence identical to E_1_ except that it was acquired in violation of the standard procedures of evidence management. Despite being physically identical, the court would prefer E_1_ to E_2_, because although both avert certain epistemic risks in virtue of their publicity, E_1_ averts additional important epistemic risks in virtue of the manner it was acquired. In other words, although physically identical, the court would consider E_1_ more objective than E_2_. Thus, in science and law, we refer to evidence as objective due to i) its public nature and/or ii) the epistemic-risk-mitigating processes whereby we obtain it.

## 5 Another word from our skeptics

At this point we should return to the deflationary skeptical attacks on objectivity we mentioned at the outset, to see whether we've done enough to answer them. We consider our answer to MacKinnon in §4.5 sufficient. We acknowledge the validity of her claim that objectivity can function as a tool of oppression, but we don't think it necessarily must. Moreover, we see authors like MacKinnon as allies in the process of managing epistemic and phronetic risks. Such critics highlight collective and structural biases at work in the law that we must manage if we hope to have an objective and just legal system. Far from deflating objectivity, then, such critics do important work to make it possible.

What about Hacking's deflationary worries? Does the term ‘objectivity’ really do any work on our approach? In other words, does talk about objectivity do anything over and above simply pointing to the totality of risks discussed in section four? Why talk about objective evidence, for example, rather than simply saying that in selecting and gathering our evidence we've managed the known epistemic risks? Why talk about deliberative objectivity rather than saying that normatively correct deliberative procedures were followed? Why not just stop talking about objectivity and focus instead on doing ground-level analysis of the specific epistemic virtues and vices in individual instances of scientific research, as Hacking suggests? What is the umbrella term “objectivity” actually good for beyond generating confusion and disagreement? Are we stuck with the term simply because scientific and legal discourse is shot through with talk about objectivity? Is that sufficient reason to preserve an idle wheel in our epistemic and phronetic practices?

We reject Hacking's scepticism on two grounds. First, on Hacking's own terms it matters normatively that objectivity is doing work in our knowledge acquisition practices. Hacking, following J.L. Austin (1961), enjoins us to examine terms in their “sites of use” (pp. 14–15), and he appeals to the case of the ‘Commission of Inquiry into the Decline of Sockeye Salmon in the Fraser River’ as evidence that the concept of objectivity is not used in practice. However, we argue that objectivity allows us to maintain the division of epistemic labour. That is, when we look to its sites of use we see that it is not merely some esoteric philosophical concept, but rather that it forms part of a socially useful practice. And on Hacking's Austin-inspired account, that a term *is* used in practice provides us with good reason to use it in philosophy.

Secondly, we can also find some guidance for the usefulness of the abstract concept of objectivity in an analogous debate in political philosophy over the usefulness of the concept of ‘justice’ – this is typically referred to as the debate between ‘ideal’ and ‘non-ideal’ theory. Amartya Sen (2006), for instance, argues that we do not need an ideal theory of justice in order to pursue actual justice in the real world, and that the ideal theory can even be a hindrance to this practical work. By contrast, John Rawls (1999), argues that we need the ideal theory so that we have something to aim at (pp 89–90). While it is beyond the scope of this paper to engage fully in debate over ideal versus non-ideal theories of justice (see Laura Valentini (2012) for a more complete articulation of this debate), this at least shows us the appeal of abstract philosophical concepts, such as justice and objectivity. Consider, for instance, only being able to point to individual instances of social wrongs in the world typically associated with injustice; we could, for example, identify cases of police brutality and say that something socially bad has happened there, but it would still be useful to appeal to the abstract concept of injustice to explain that badness. Similarly, while we could point to particular instances of scientific research vice (as Hacking recommends), such as bias, it would still be useful to be able to refer to the ideal of objectivity to explain what is going wrong when these vices occur.^16^ Our commitment to justice as a normative ideal keeps us vigilant in the fight against known manifestations of injustice; but it also keeps us on the lookout for a) hitherto unknown tokens of known types of injustice and b) unknown types of injustice; and when we find these unknown tokens and types it affords us an abstract concept to appeal to as we make sense of and learn how to manage them. Our commitment to objectivity serves a similar role our attempts to get things right, promoting vigilance in our efforts to manage known epistemic and phronetic risks, keeping us on the lookout for new tokens and types of such risks, and helping us make sense of and manage these new tokens and types as they arise. As an organising ideal for our practices, objectivity, like justice, does important work.

## 6 Conclusion

Taking inspiration from Koskinen's (2018) recent work, this paper has proposed a shared rescue strategy for objectivity in science and the law. In science, we need assurance that we can rely on each other as epistemic agents, which requires guarding against a variety of very specific epistemic risks in very context-specific ways. Objectivity is the assurance that such epistemic risks have been managed. This is Koskinen's argument. We argue that the same is true in the law. The law's legitimacy rests on its claim to objectivity. In other words, to be legitimate, the law needs to manage a range of epistemic, ethical and phronetic risks. We argue that legal objectivity is the assurance that context-specific measures have been taken to manage such risks, and we have provided an indicative list of what some of the objectivity-assuring strategies in the law currently are or should be. This list is not exhaustive, and it will doubtlessly change as new sources of epistemic and phronetic risks in the law emerge.

We believe that this approach to objectivity in the law can move us beyond the dialectical stalemate in the current conversation about objectivity. Critics argue that genuinely determinate and objective legal judgments are utterly beyond our reach, and so we should abandon all pretence of the law's objectivity. Defenders of objectivity insist that the ideal is indispensable, but their insistence on conceptualizing objectivity as determinacy fails to do justice to the legitimate concerns raised by the critics of objectivity, and so tends to alienate them. The risk account of objectivity, we believe, can satisfy disputants on both sides of this divide. For it acknowledges the power of the best critiques of objectivity; indeed, it sees these critiques as an essential part of the objective stance, as they identify the very epistemic and phronetic risks that objective legal practice must manage. But the risk account of objectivity also does justice to the fact that the law is saturated with the language of objectivity and that the ideal does important work in practice. We are hopeful, then, that this approach will help us make progress in contemporary debates about objectivity in science, law, and in the domains where such concerns intersect, e.g., the intersection of law and psychiatry. If we listen to the voices of all concerned, and take seriously the epistemic and phronetic risks they call to our attention, a workable approach to objectivity might just be within our fallible, imperfect human reach.

## Footnotes

1 Members of the Essex Autonomy Project presented these findings in February of 2017 at the Policy Institute at King's College for the first event associated with the *Mental Health and Justice* project, a multi-disciplinary research initiative funded by the Wellcome Trust. Wayne Martin, Matt Burch, and Sándor Gurbai prepared the public policy lab on objectivity.

2 Cited in Miller (2017). The *Institutes of Justinian* is a codification of Roman Law from 6th c. AD.

3 For a lucid discussion of the intellectual virtue of phronēsis, see McMullin (2018, pp. 110–114).

4 Indeed, in most cases the law must avert many phronetic risks as once. For instance, in the child protection cases that Munro and Hardie (2018) in their recent work on objectivity, when the Family Court makes assessments about whether an at-risk child should remain with their family or be placed in care, the Court must simultaneously manage risks that have to do with our limited abilities to forecast future outcomes, the social value we place on keeping families intact, the potential physical harms to the child, and to treating all the relevant parties (parents, the child, other caregivers) with appropriate concern, amongst other risks.

5 From here on, when we refer to important epistemic/phronetic risks, readers can assume that we mean important epistemic/phronetic risks that arise from our imperfections as epistemic agents/practical reasoners.

6 “Context” in the law means that what will be relevant will vary on a case-by-case basis. Philosophy of science typically draws a distinction between the context in which knowledge is *produced* and the context in which it us *used* (Montuschi, 2016, 60), but given the deeply applied nature of the law, all contexts are contexts of use.

7 Parascandola goes on to argue that p-value standardisation creates additional risks, and so is ultimately not successful. But our point was to highlight how scientists use interpretative norms to manage epistemic risks, not to assess the value of a particular standard.

8 This remark is cited in Guest (2007, p. 100) and comes from Susskind (2005).

9 Of course, as Goldman also points out, not all evidence-handling procedures aim at truth. For instance, exclusionary rules make illegally obtained evidence inadmissible, even though such evidence might lead to a more truthful determination of the facts. This does not, however, make the law less objective. It just highlights the fact that strategies designed to manage epistemic risks must simultaneously manage relevant ethical and phronetic risks. Courts do not admit illegally obtained evidence because of the associated ethical risks, e.g., doing so might incentivize criminal behavior amongst people trying to win a case. However, in defining what constitutes illegally obtained evidence, the law also has to manage phronetic risks, e.g., the risk of making the wrong call when balancing the value of a more truthful determination of the facts against the ethical risks just mentioned.

11 For discussions of the problem of conflicts of interest in relation to objectivity see Harding (1992) or Resnik (1998).

12 See Charlton, 1998.

13 Miranda Fricker's *Epistemic Injustice* (2007) shows that exclusions of the views of members from marginalised groups might not just be an epistemic loss, but may also be an injustice to those excluded. And so even focussing just on the epistemic may sometimes also involve considerations about justice.

14 Relevantly different here means that a person is different in such a way that treating her like everyone else would constitute unfair treatment, e.g., failing to provide a person with intellectual difficulties with reasonable accommodations in her education would constitute a failure to treat differently someone who is relevantly different.

15 For Conee and Feldman (2008) scientific evidence is “publicly available” by definition (p. 84). We are not sure that all scientific evidence must be publicly available—after all, introspective reports are private, yet they still serve as evidence in psychological science. But we agree that publicly available evidence is especially important in science.

16 Jack Wright (2018) makes a similar point, arguing that objectivity is a ‘relational term’, by which he means that “objectivity helps to relate practices to other practices and to the abstract uses, goals, ideals, and types of practices associated with types of objectivity” (p. 389). Thinking of Wright's suggestion as analogous to the debate between ideal versus non-ideal theory (as described above) is useful, just because of how much material that debate has produced, thus giving an idea of what the relational concept might look like.

## Footnotes

10 This is only part of Waldron's list but we thought it sufficient to make our point.
